# Dual tree complex wavelet transform-based signal denoising method exploiting neighbourhood dependencies and goodness-of-fit test

**DOI:** 10.1098/rsos.180436

**Published:** 2018-09-19

**Authors:** Khuram Naveed, Bisma Shaukat, Naveed ur Rehman

**Affiliations:** Department of Electrical Engineering, COMSATS University Islamabad (CUI), Park Road, Islamabad, Pakistan

**Keywords:** signal denoising, goodness-of-fit test, dual tree complex wavelet transform, translation invariance, neighbourhood filtering

## Abstract

A novel signal denoising method is proposed whereby goodness-of-fit (GOF) test in combination with a majority classifications-based neighbourhood filtering is employed on complex wavelet coefficients obtained by applying dual tree complex wavelet transform (DT-CWT) on a noisy signal. The DT-CWT has proven to be a better tool for signal denoising as compared to the conventional discrete wavelet transform (DWT) owing to its approximate translation invariance. The proposed framework exploits statistical neighbourhood dependencies by performing the GOF test locally on the DT-CWT coefficients for their preliminary classification/detection as signal or noise. Next, a deterministic neighbourhood filtering approach based on majority noise classifications is employed to detect false classification of signal coefficients as noise (via the GOF test) which are subsequently restored. The proposed method shows competitive performance against the state of the art in signal denoising.

## 1. Introduction

Noise corrupting signals during their acquisition and transmission is a well-known phenomenon. The processing of such noisy signals, in real-world applications, requires their denoising as an essential preprocessing step. Assume that **s** denotes a true signal which is corrupted by additive noise ***η*** to yield its noisy observation **x** as follows:

1.1 x=s+η.

The goal of signal denoising is to estimate the true signal from its noisy version. The noise ***η***, for instance, could be assumed to be belonging to the Gaussian distribution $N(0,\;\sigma^{2})$ with zero mean and arbitrary variance *σ*^2^.

Wavelet transform presents itself as one of the most effective tools for signal denoising due to its sparse signal representation at multiple scales. Wavelet decomposition distributes the signal discontinuities in its locality at multiple scales causing higher amplitudes for wavelet coefficients corresponding to desired signal, while multiscale noise coefficients are uniformly distributed across all scales. This sparsity within wavelet coefficients is exploited by estimating a threshold value to distinguish between the coefficients corresponding to noise and the desired signal.

In order to estimate the true signal **s** from **x**, the wavelet-based denoising schemes start by taking wavelet transform W(⋅) of the noisy signal, followed by the nonlinear thresholding operation T(⋅). Finally, signal reconstruction is achieved by taking the inverse wavelet transform $W^{-1}(\cdot )$

1.2 $$\hat{s}=W^{-1}(T(W(x))),$$

where $\hat{s}$ denotes the estimate of the true signal **s**.

Traditionally, discrete wavelet transform (DWT) has been used efficiently, in combination with a simple nonlinear thresholding operation on the resulting wavelet coefficients, to resolve the problem of signal denoising [1]. The seminal hard and soft thresholding operations introduced by Donoho & Johnstone [2,3] paved the way for various DWT-based denoising algorithms. *VisuShrink* [4] employs a universal threshold for all the wavelet coefficients, which is computed by taking into account the noise variance *σ*^2^ and the signal length *N* as

1.3 $$T_{u}=\sqrt{2\sigma^{2}\log N}.$$

Contrarily, the *SureShrink* [5] performs denoising by employing adaptive threshold for wavelet coefficients at multiple scales via Stein's unbiased risk estimator (SURE). A few denoising approaches based on empirical Bayes [6–8] also exploit the sparsity of the wavelet transform, where the level of sparseness of wavelet coefficients is estimated by maximizing a marginal log-like cost function. The statistical dependencies between the wavelet coefficients have also been explored as an avenue for signal denoising in [9–11].

Maximal decimation in DWT accounts for aliasing in wavelet coefficients resulting in the loss of translation invariance within the coefficients. Subsequently, the inverse-DWT filters are designed to overcome aliasing but only if the wavelet coefficients remain unchanged. However, in DWT-based signal denoising [4], wavelet coefficients are altered during the thresholding operation causing various unwanted artefacts in the denoised signal. A possible solution would be the decimation-free DWT but its maximal redundancy makes it computationally inefficient.

**Figure 1.**
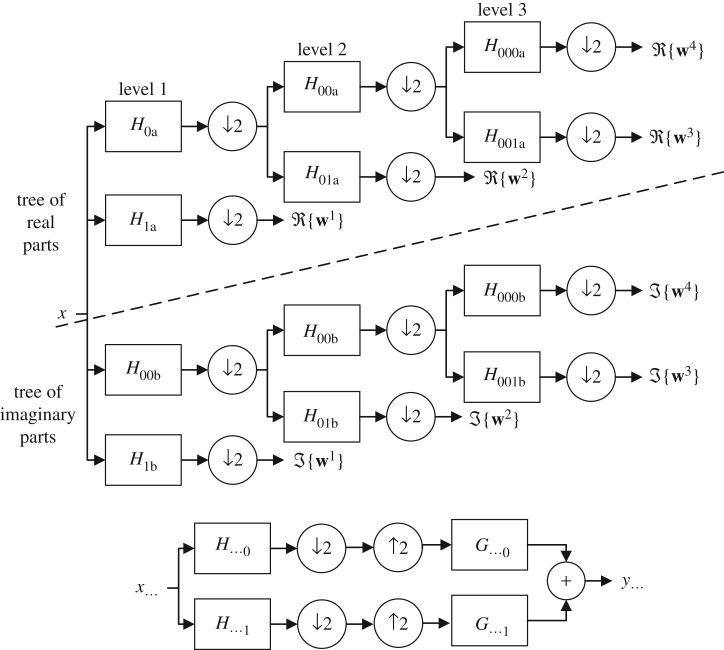
Depiction of the dual tree of wavelet filters employed by the DT-CWT to decompose a signal into real and imaginary parts of the complex wavelet coefficients separately.

To resolve this issue, an enhanced version of DWT, namely the dual tree complex wavelet transform (DT-CWT), has recently emerged, providing an enhanced platform for denoising and other applications owing to its quasi-translation invariance property for decomposed coefficients [12]. The DT-CWT decomposes a signal into complex wavelet coefficients via dual tree of wavelet filters [13] as shown in figure 1, whereby both real and imaginary parts of the coefficients are computed via a separate tree of filters which are independent to each other. Consequently, both real and imaginary parts of complex wavelet coefficients are dealt with as independent sets of wavelet coefficients, making the DT-CWT twice as redundant as the DWT [13]. This 2 : 1 redundancy helps reduce aliasing in the DT-CWT coefficients [14], making it robust to artefacts. The authors [15–17] demonstrate that better shift invariance and reduced spectral aliasing enables the DT-CWT-based denoising methods to perform better than the conventional DWT. Therefore, DT-CWT has been used for suppressing noise in a variety of real-world signals, i.e. ECG signal denoising [18], seismic signal denoising [19], SAR despeckling [20] and medical image denoising [21]. Mathematically, the DT-CWT decomposition of an input noisy signal **x** at multiple scales is given as follows:

1.4 $$w^{k}=W_{d}(x),$$

where the forward DT-CWT operation is denoted by $W_{d}$ resulting in the vector of complex wavelet coefficients **w**^*k*^ at *k*th scale. Since, real and imaginary parts of DT-CWT coefficients are stored and processed as independent wavelet coefficients, we, respectively, denote them by ℜ{**w**^*k*^} and ℑ{**w**^*k*^} in the rest of the paper.

Classic DWT- or DT-CWT-based multiscale noise shrinkage strategies operate on individual wavelet coefficients by comparing them against a threshold value for signal/noise detection. On the other hand, sufficient literature is also available on neighbourhood-based multiscale denoising strategies [22–25]. These methods exploit the fact that the wavelet coefficients corresponding to signal discontinuities lie in the neighbourhood of each other and have higher amplitudes compared with the wavelet coefficients corresponding to noise [22]. Hence, deterministic as well as statistical characteristics of the neighbourhood of wavelet coefficients belonging to signal must be vastly different from the neighbourhood of noisy coefficients. This fact motivates various denoising strategies which exploit deterministic or statistical neighbourhood dependencies of multiscale wavelet coefficients.

Cai & Silverman [22] proposed two neighbourhood-based noise shrinkage algorithms for one-dimensional signals, namely *NeighCoeff* and *NeighBlock*, which exploit deterministic neighbourhood dependencies in order to make decision regarding the presence of noisy coefficients. *NeighShrink* [23] extends the idea of noise shrinkage based on deterministic neighbourhood dependencies to the two-dimensional signals (images), whereby a sum of squared neighbouring coefficients, *S*^2^_*i*,*j*_, is compared against the square of universal threshold *T*_u_^2^. If *S*^2^_*i*,*j*_ is less than *T*_u_^2^, then the central coefficient *d*_*i*,*j*_ is considered as noise and is set to zero, else, a customized soft thresholding is applied on the central coefficient via the following relation:

1.5 $$d_{\;j,k}=d_{\;j,k}\left(1-\frac{T_{u}^{2}}{S_{i,j}^{2}}\right).$$

*NeighSure* [24] exploits the near translation invariance of the DT-CWT by extending the neighbourhood-dependent thresholding to the dual tree of complex wavelet coefficients whereby adaptive threshold was employed based on SURE. Another denoising strategy employs similar neighbourhood filtering on the complex DT-CWT coefficients in [25].

Among other multiscale approaches for signal denoising an empirical mode decomposition-based denoising approach has been proposed in [26] which employs nonlinear thresholding similar to the DWT-based denoising approaches. Recently, multivariate extensions of EMD [27] have also been employed for signal denoising [28]. An efficient algorithm based on total variation filtering for denoising (TVD), which happens to be very fast and non-iterative, has been reported in [29]. Similarly, total generalized variation based denoising method [30] has also been reported in the literature.

Recently, statistical tools like the Bayesian local false discovery rate (BLFDR) and the local goodness-of-fit (GOF) test have been employed for multiscale noise shrinkage, which exploit statistical neighbourhood dependencies of wavelet coefficients. The BLFDR-based shrinkage [31] defines denoising in terms of Bayes factors (hypothesis testing), whereby local or neighbourhood-based Bayesian false discovery rate is estimated to identify noisy coefficients. In our previous work, the GOF test-based denoising framework namely *TI-DWT-GOF* [32] exploits statistical neighbourhood dependencies based on empirical distribution function (EDF) statistics for identification of signal/noise coefficients.

Despite exhibiting comparative state-of-the-art performance, the *TI-DWT-GOF* suffers from two major limitations: (i) lack of translation invariance in the DWT coefficients, resulting in the artefacts in the denoised signals and (ii) the trade-off between probability of false alarm and probability detection was not considered while selecting the optimal threshold value, which lead to the false detection of several signal coefficients as noise, causing loss of signal details. This paper introduces a novel signal denoising framework^1^ which effectively compensates for these shortcomings via the following two enhancements:

(i) Nearly translation invariant DT-CWT is employed (instead of the DWT) for decomposition of noisy signal into multiple scales followed by the GOF-based classification of complex wavelet coefficients as signal or noise. Note that the proposed extension of the GOF test on DT-CWT coefficients is not trivial due to the following reasons: (a) it involves computation of the thresholds (as a function of *P*_fa_) separately for the real and imaginary DT-CWT wavelet coefficients; (b) a parallel framework is designed to independently perform the proposed GOF-based thresholding operation on the real and imaginary parts of the complex wavelet coefficients.
(ii) A novel post-processing filtering approach, based on majority noise classifications in a neighbourhood of the wavelet coefficient being considered, is introduced to recover wrongly classified signal coefficients as noise by exploiting the deterministic neighbourhood dependencies within DT-CWT coefficients.

Novel contribution of the proposed method includes the introduction of a novel neighbourhood filtering-based post-processing step for minimizing artefacts occurring due to false classifications of signal coefficients as noise (and vice versa) and then reverse them. In addition, a bivariate extension of the GOF-based noise detection procedure to real and imaginary coefficients of DT-CWT is proposed which exploits the near translation invariance of the DT-CWT for improved denoising performance.

The GOF test for preliminary classification of noisy wavelet coefficients employs Anderson–Darling (AD) statistics based on EDF as a tool to measure the similarity between the complex wavelet coefficients and the reference Gaussian noise distribution. Since the GOF test is basically a hypothesis testing tool, we devise preliminary GOF-based classification/detection as a hypothesis testing problem where the null hypothesis, denoted by $H_{0}$, corresponds to the detection of noise while the alternative hypothesis, denoted by $H_{1}$, corresponds to the detection of signal. Next, a novel neighbourhood filtering-based post-processing step is employed in the vicinity of coefficients classified as noise, whereby true noise coefficients are distinguished from the ones falsely classified as noise by investigating whether their neighbourhoods contain majority noise classifications or otherwise.

The composition of rest of this paper is as follows: §2 presents the background of GOF testing, while §3 presents the proposed algorithm. Section 4 discusses the simulation results of the proposed algorithm against the state of the art in signal denoising on synthetic as well as real signals. At the end, §5 concludes the article while also highlighting avenues for future research work.

## 2. Background

The statistical GOF testing is used to check how well a specified model or distribution fits a given set of observations. In the GOF tests, a statistical measure is employed to quantify the difference between the observed values and the specified or reference values. Next, hypothesis testing is performed by comparing the computed measure in the previous step against the threshold *T* which is a function of probability of false alarm (*P*_fa_). Traditionally, the GOF tests have been used for spectrum sensing applications [34,35], however, recently these have been applied on multiscale data for signal denoising in [32,36].

There are multiple choices of quantitative measures to perform the GOF testing; however, only AD statistics will be discussed here. The AD measure employs the statistics based on the empirical cumulative distribution function (ECDF) to quantify the distance between two set of observations. Let the ECDF of the local wavelet coefficients under observation be denoted by F(x), and CDF of the reference Gaussian noise is denoted by $F_{r}(x)$, then the AD statistics measure *τ* for distances between the two CDFs is defined mathematically as

2.1 $$\tau =\int_{-\infty }^{\infty }{\left(F_{r}(x)-F(x)\right)}^{2}\psi (F_{r}(x))\;\text{d}(F_{r}(x)),$$

where $\psi (F_{r}(x))$ is a non-negative weighting function, defined as $\psi (F_{r}(x))=(F_{r}(x){\left(1-F_{r}(x)\right)}^{-1}$ over the interval 0 ≤ *x* < 1. The non-negative weighting function $\psi (F_{r}(x))$ in AD statistics is designed to give more weight to the tail of the distribution, making AD statistics a robust and flexible measure. If the given dataset is divided into segments of length *l*, a convenient numerical expression for AD statistics is written as

2.2 $$\tau =l-\sum_{n=1}^{l}\frac{\left(2i-1\right)}{L}(\text{ln}(F_{r}(x_{i})-\text{ln}(F_{r}(x_{L+1-i}))),$$

where probability distributions are asymptotically defined if l→∞.

A threshold *T* is selected as a function of error rate for which candidate distribution is falsely rejected. This error rate is termed as probability of false alarm (*P*_fa_), which refers to the probability of erroneously detecting a noise sample as one from the desired signal. Mathematically, the *P*_fa_ can be defined as follows:

2.3 $$P_{\mathrm{fa}}=\text{Prob}\{\tau >T|H_{0}\}.$$

For better denoising results, it is desirable that *T* is selected by minimizing the *P*_fa_.

In the GOF tests, the hypothesis testing is performed by comparing *τ* against *T*. If *τ* < *T*, the observed signal is considered to be originated from the reference noise distribution, i.e. noise is detected, otherwise desired signal is detected.

The existing GOF-based denoising framework, i.e. *TI-DWT-GOF* introduces the use of GOF test on multiscale data obtained by operating the DWT on a noisy signal [32]. To this end, EDF statistics-based statistical distance is estimated between the multiscale coefficients and the noise distribution, which is then compared against a threshold value. The threshold represents the upper bound of the EDF-based statistical distance for noise coefficients, which is estimated by minimizing the *P*_fa_. The lack of translation invariance in the DWT is partially compensated by the cycle spinning approach [37].

## 3. Proposed algorithm

The proposed method couples the local statistical GOF test with a novel neighbourhood filtering-based majority classifications to formulate a robust two-step procedure for signal denoising. The proposed framework employs near translation invariant multiscale decomposition of noisy signal through the DT-CWT which aims to suppress the undesired artefacts otherwise present in the DWT-based denoising results.

The lack of translation invariance in the DWT is caused by maximal decimation, where spectral aliasing becomes inevitable once the wavelet coefficients are perturbed (during the thresholding operation in denoising), causing undesired artefacts in the denoised signal. On the contrary, translation invariance is approximately preserved in the DT-CWT coefficients due to the following reasons: (i) the complex representation of DT-CWT coefficients renders the Fourier transform (FT)-like properties to the DT-CWT (i.e. FT is translation invariant) [14]; (ii) the separate trees of wavelet filters for independent computation of the real and imaginary parts of complex wavelet coefficients in DT-CWT results in redundancy, thereby reducing the effect of maximal decimation (performed in both trees of decomposed coefficients) [14]. Hence, the alteration of the DT-CWT coefficients during the thresholding operation does not result in major artefacts in the denoised signal. This fact advocates the choice of the DT-CWT as a part of the proposed denoising framework which is a significant enhancement to the already existing GOF-based signal denoising framework *TI-DWT-GOF*.

Owing to the unavailability of a generalized distribution supporting all kind of noise-free signals, empirical computation of the probability of detection (*P*_d_) (i.e. probability of falsely detecting signal coefficients as noise) is not possible. Consequently, the trade-off between the *P*_fa_ and the *P*_d_ is not considered in the threshold estimation process in the *TI-DWT-GOF* and the proposed method. Instead, thresholds are estimated by minimizing the *P*_fa_ of detecting noise coefficients as signal alone. This leads to various false detections of signal coefficients as noise in the *TI-DWT-GOF* resulting in deteriorated denoising performance. To counter this issue, the proposed method employs a novel majority classifications-based neighbourhood filtering as the post-processing step which seeks to recover the false noise detections by the GOF test; this post-processing step is denoted as *NeighFilt*.

The proposed algorithm can be explained by dividing into two main parts: GOF-based preliminary classification of multiscale coefficients and majority classification-based neighbourhood filtering. Each part is explained in detail below.

### 3.1. GOF test-based preliminary classification

**Figure 2.**
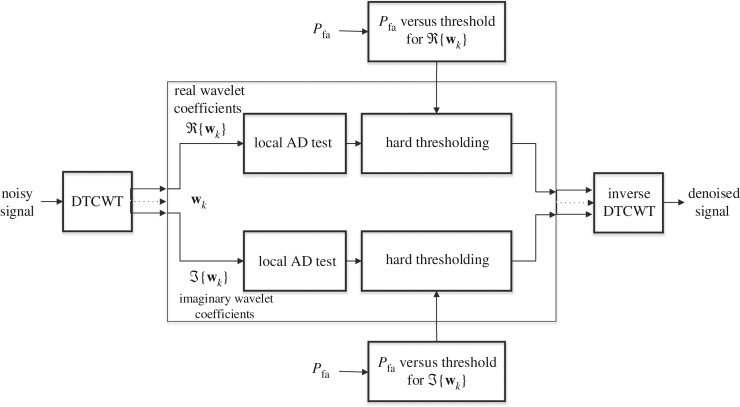
Block diagram of the proposed denoising method.

In our proposed method, the GOF test based on EDF statistics has been used to distinguish between the wavelet coefficients corresponding to noise and desired signal. AD measure based on EDF statistics is used wherein the distance *τ* between the EDF of a given set of wavelet coefficients and the EDF of the reference noise distribution is empirically estimated via equation (2.2). It should be worth mentioning that the choice of AD statistics in this work is due to its robustness and flexibility over other test statistics based on EDF [38]. Later, the AD measure *τ* is compared against a threshold value *T* which is estimated as a function of *P*_fa_. The procedure of GOF test-based preliminary classification of noisy coefficients is explained through block diagram in figure 2. It should be noted that, in order for the GOF test to work, prior knowledge of the reference noise distribution is a must. While the proposed framework has potential to detect and remove any kind of noise provided its distribution model is known *a priori*, the scope of this work is only limited to Gaussian noise distribution. Therefore, we consider reference noise distribution as additive white Gaussian noise (AWGN) with zero mean and arbitrary variance *σ*^2^, i.e. $N(0,\sigma^{2})$. Alternatively, the reference noise distribution could have been estimated from input data at hand, but that would present a very challenging scenario and is not considered in this work. In our method though, while we chose zero mean Gaussian distribution as our reference, we estimate the noise variance from input data and show that our method is robust in such cases.

Moreover, since the proposed method uses GOF test to compare between the reference and the empirical input data distribution, difference between the reference and ‘observed’ noise distributions is bound to cause false detections (noise falsely detected as data).

Here, the preliminary detection of the noisy coefficients is formulated as a hypothesis testing problem via the GOF test, whereby the null hypothesis $H_{0}$ corresponds to the detection of noise and the alternative hypothesis $H_{1}$ accords with signal detection. To achieve that, the signal first needs to be decomposed at multiple scales before the use of the GOF tests. Hence, the operation of GOF tests on wavelet coefficients for binary hypothesis testing is defined as

3.1 $$H_{0}:\tau \leq T^{k}\;\text{and}\;H_{1}:\tau >T^{k},$$

where $H_{0}$ denotes the case when local wavelet coefficients are detected as noise, while $H_{1}$ denotes the case when these coefficients are detected as desired signal. The discussion on the procedure adopted by the proposed method is divided into two main steps, namely the threshold estimation and the nonlinear thresholding operation.

#### 3.1.1. Threshold estimation

**Figure 3.**
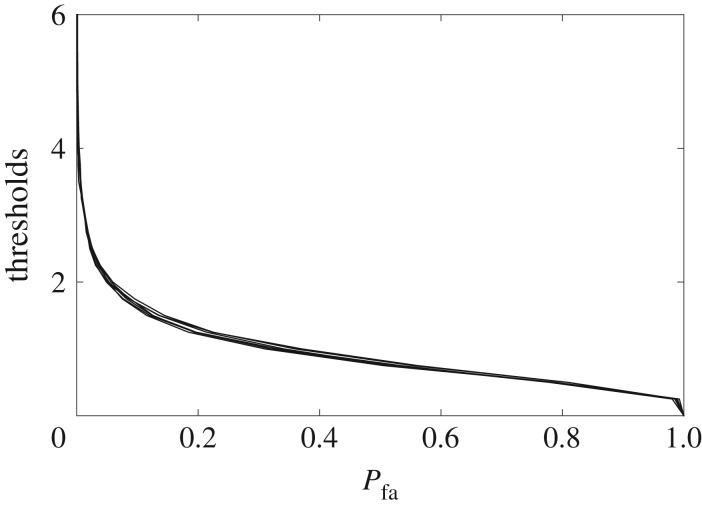
Thresholds *T*^*k*^ versus *P*_fa_ graphs plotted together for Gaussian noise at multiple scales.

In the literature related to spectrum sensing, multiple tables for threshold *T* versus *P*_fa_ are available for performing the GOF tests [38,39]. These tables can be of relevance to multiscale signal denoising because the linear operations in DT-CWT do not alter the distribution of noise. However, an alternative numerical approach based on repeated simulations on large realizations of noise ***η*** (Gaussian noise in our case) is adopted for threshold estimation here. For that purpose, *J* = 1000 realizations of ***η***, each of length *l* = 1000, were decomposed via DT-CWT, at *k* = 1 … *K* scales, to obtain the noisy (complex) wavelet coefficients **w**^*k*^_*η*_ whose real parts are denoted by ℜ{**w**^*k*^_*η*_} and imaginary parts are denoted by ℑ{**w**^*k*^_*η*_}. In both cases, the reference Gaussian CDFs are, respectively, denoted by $F_{ℜ}^{k}(x)$ and $F_{ℑ}^{k}(x)$ at each scale *k*. Afterwards, AD statistics measures *τ*^*k*^_ℜ_ and *τ*^*k*^_ℑ_ were computed separately for each of the real and imaginary trees of wavelet coefficients by considering $F_{r}(x)=F_{ℜ}^{k}(x)$ or $F_{ℑ}^{k}(x)$, as the case may be. Finally, threshold *T*^*k*^ versus *P*_fa_ graphs were obtained for each scale by employing equation (3.1) on ℜ{**w**^*k*^_*η*_} and ℑ{**w**^*k*^_*η*_} separately followed by the selection of thresholds *T*^*k*^_ℜ_ and *T*^*k*^_ℑ_ for a given value of *P*_fa_. This procedure was repeated for all scales and the resulting threshold versus *P*_fa_ tables are plotted in figure 3.

It was observed that the empirically estimated reference distributions at each scale preserved the Gaussianity in the noisy wavelet coefficients, owing to the linear nature of DT-CWT operations. For this reason threshold *T*^*k*^ versus *P*_fa_ graphs were found to be similar for all scales. The above procedure for selecting threshold *T*^*k*^ for a given value of *P*^(*k*)^_fa_ at each scale *k*, can be expressed as follows:

3.2 $$T^{k}=T(w^{k},\;P_{\mathrm{fa}}^{\left(k\right)}),$$

where T denotes the threshold estimation process explained above.

#### 3.1.2. GOF-based thresholding operation

Once the thresholds for each scale are selected, they are applied on wavelet coefficients through the hard thresholding operation. Typically, in thresholding, the wavelet coefficients of noisy signal are compared against the threshold value, whereby the coefficients below the threshold level are identified as noise and are set to zero. However, the GOF testing-based thresholding does not involve any direct comparison of threshold and signal coefficients at multiple scales. Instead, it performs hypothesis testing via equation (3.1). In this scheme, the thresholds *T*^*k*^ are compared against the AD statistics measure *τ* computed locally for wavelet coefficients **w**^*k*^, if *τ* < *T*^*k*^ (i.e. $H_{0}$) the local wavelet coefficients are recorded as noise and replaced by zero. On the other hand, if *τ* ≥ *T*^*k*^, i.e. ($H_{1}$), the coefficients are retained as desired signal. In this work, two sets of thresholds, i.e. *T*^*k*^_ℜ_ and *T*^*k*^_ℑ_ are employed to independently perform thresholding operation on ℜ{**w**^*k*^} and ℑ{**w**^*k*^} at each scale *k*.

The procedure involved in GOF-based thresholding operation is divided into multiple steps, which are discussed next in detail. Firstly, the variance of noise *σ*^2^ is estimated using Donoho's robust median estimator [40], as

3.3 $$\sigma =\frac{\text{Median}(ℜ\{w^{1}\})}{0.6745}.$$

Next, the normalization of the wavelet coefficients by *σ* is performed as

3.4 $${\tilde{w}}^{k}=\frac{w^{k}}{\sigma },$$

where the notation ${\tilde{w}}^{k}$ is adopted for generality which encompasses both real and imaginary normalized wavelet coefficients, $ℜ\{{\tilde{w}}^{k}\}$ and ℑ$\left\{{\tilde{w}}^{k}\right\}$.

The GOF tests-based thresholding is applied locally on the normalized wavelet coefficients ${\tilde{w}}^{k}$ at all scales. Here, the GOF thresholding operation, denoted by G, operates in parallel on both trees of wavelet coefficients $ℜ\{{\tilde{w}}^{k}\}$ and ℑ$\left\{{\tilde{w}}^{k}\right\}$. Firstly, the AD statistics *τ*^*i*^_ℜ_ and *τ*^*i*^_ℑ_ are calculated for small segments around the *i*th coefficients $ℜ\{{\tilde{w}}^{k}\}(i)$ and ℑ$\left\{{\tilde{w}}^{k}\}(i\right)$, respectively, via equation (2.1). The *τ*^*i*^_ℜ_ and *τ*^*i*^_ℑ_ are then compared against the respective thresholds *T*^*k*^_ℜ_ and *T*^*k*^_ℑ_ by employing equation (3.1) for testing the hypothesis that the coefficients under observation namely $ℜ\{{\tilde{w}}^{k}\}(i)$ and $ℑ\{{\tilde{w}}^{k}\}(i)$ belong to noise or the desired signal. In the former case, the coefficient is set to zero while coefficient is retained in the latter case. Mathematically, the process can be represented as

3.5 $${\hat{w}}^{k}=G({\tilde{w}}^{k},\;T^{k}),$$

where G denotes the GOF thresholding operator which employs hypothesis testing equation (3.1) to either retain or remove wavelet coefficients, ${\hat{w}}^{k}$ based on the threshold *T*^*k*^.

**Figure 4.**
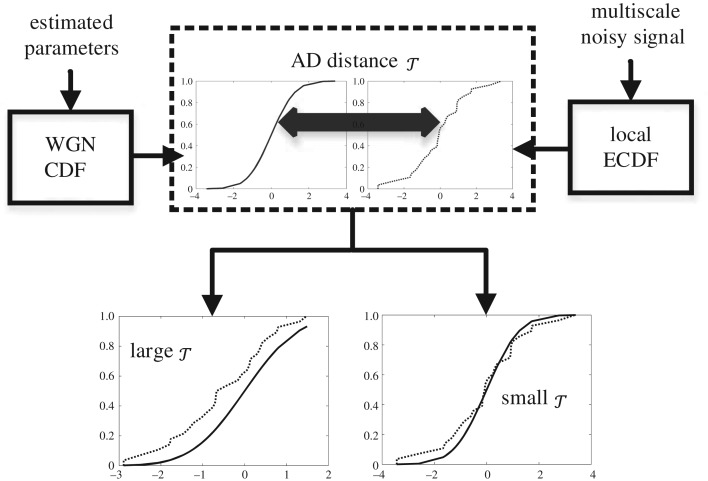
Computation of AD statistical distance *τ* between ECDF of the local wavelet coefficients and CDF of reference WGN: (lower left) an example where CDFs are different, yielding large value of *τ* resulting in detection of desired signal; (lower right) an example of noise detection due to smaller value of *τ* hinted by the close similarity of the two CDFs.

An illustration of the signal and noise classification via the GOF operation is shown in figure 4. The process of computing distance between reference CDF and the ECDF of multiscale wavelet coefficients is graphically depicted inside the dotted box whereby the solid horizontal two-sided arrow denotes the AD measure *τ*. Here, figure 4 (bottom left) presents the case of noise detection and figure 4 (bottom right) shows the case of signal detection. The CDF of the local wavelet coefficients in figure 4 (bottom left) is similar to reference noise CDF (i.e. smaller distance *τ*), suggesting that these coefficients originated from the WGN distribution. On the other hand, in figure 4 (bottom right), the two CDFs are widely different (i.e. larger distance of *τ*), hinting the presence of signal.

### 3.2. Majority classification-based neighbourhood filtering

The preliminary GOF-based detections of signal and noise coefficients contain several false detections of signal as noise since the trade-off between the *P*_fa_ and the *P*_d_ is not considered within the GOF test.

In order to recover those false detections, a novel neighbourhood filtering method *NeighFilt*, is proposed in this section as a post-processing step. The *NeighFilt* exploits deterministic dependencies within the wavelet coefficients by following the footsteps of the *NeighCoeff* [22], *NeighShrink* [23], *NeighSure* [24] and *DTCWTNeigh* [25], whereby a noise coefficient is detected only if it is surrounded by majority noisy coefficients.

**Figure 5.**
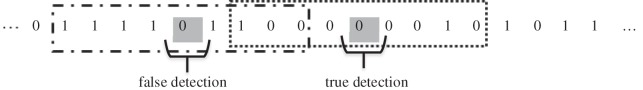
Depiction of the *NeighFilt* operation on the *mask* file where ‘0’ represents the detection of noisy coefficient via the GOF test while ‘1’ denotes the detection of signal. *False detection* is found if the noisy coefficient is surrounded by the signal coefficients and vice versa.

The *NeighFilt* checks whether a coefficient classified as noise (via the GOF test) is surrounded by the likewise noise-classified coefficients in a small neighbourhood. If so, it is considered as a *true detection* of noise coefficient as shown in figure 5 (right window) and is subsequently floored to zero. Contrarily, If a noise-classified coefficient is surrounded by majority coefficients classified as signal, then it is considered as a *false detection* as shown in figure 5 (left window), consequently, it is retained as desired signal.

Finally, the inverse DT-CWT operation $W^{-1}$ is performed on the thresholded wavelet coefficients ${\hat{w}}^{k}$ which are reverse normalized prior to the reconstruction as follows:

3.6 $$\hat{s}=W^{-1}({\hat{w}}^{k}\times \sigma ),$$

where $\hat{s}$ is the denoised signal, otherwise called the estimate of the true signal **s**.

We denote the proposed method as *DTCWT-GOF-NeighFilt* in the rest of this paper. Matlab code for the proposed *DTCWT-GOF-NeighFilt* is freely available at https://www.mathworks.com/matlabcentral/fileexchange/64577-dtcwt-gof-neighfilt.

## 4. Results and discussion

In this section, a comparison of the experimental results obtained from the proposed method against the state-of-the-art signal denoising methods is presented. To this end, we present the performance analysis of comparative methods on synthetic as well as real signals. The state-of-the-art methods selected for comparison against the proposed method include (i) *DTCWT* [14], (ii) *TI-EMD* [26], (iii) *TVD* [29] (iv) *BLFDR* [31] and (v) *TI-DWT-GOF* [32]. The quantitative measures such as signal-to-noise ratio (SNR) and mean squared error (MSE) have been employed to specify the performance of the signal denoising methods.

A detailed experiment involving several segment lengths *l* in the GOF tests was also conducted to choose the optimal segment lengths *l* for denoising purpose. It was observed that the denoising results were not very sensitive to different segment lengths *l* = 14, 21, 28, 35; hence, the segment length was set to *l* = 28. The value of the probability of false alarm for the proposed method was set to *P*_fa_ = 0.005.

The dual tree of filters employed for decomposing a noisy signal into complex wavelet coefficients are taken from [41], each of length 10, which were developed by Kingsbury [42]. It was seen experimentally that the denoising results were approximately similar at the decomposition levels *K* = 3, 4, 5 and 6. We choose the number of decomposition levels to be *K* = 5 for all methods to give a fair comparison. All the other parameters relevant to the state-of-the-art methods were chosen as suggested by the authors in their respective papers.

The statistical significance of the best performing method at 5% significance level (*α* = 0.05) against the second best was verified using Student's *t*-test. The null hypothesis corresponded that the mean of the output SNR values of all realization was equal for both (best and the second best) methods implying no statistical significance, while difference in the mean (alternative hypothesis) corresponded to statistical significance.

### 4.1. Experimental results on synthetic signals

**Figure 6.**
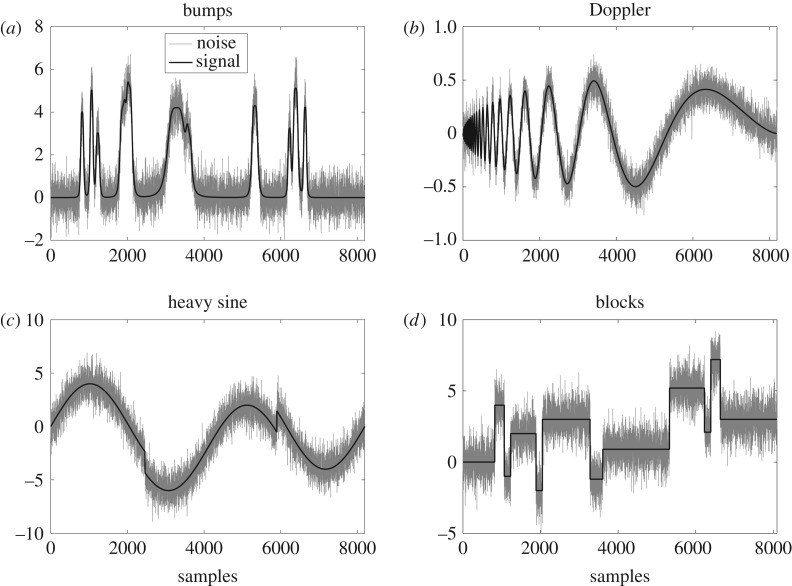
Input test signals employed for comparing the performance of proposed method against the state-of-the-art methods. (*a*) Bumps, (*b*) Doppler, (*c*) heavy sine and (*d*) blocks.

In our experiments, standard test signals including ‘bumps’, ‘blocks’, ‘heavy sine’ and ‘Doppler’ were employed. The noise corrupted versions of the aforementioned test signals with length *L* = 2^13^ and SNR = 10 dB are shown in figure 6 where the signals can be seen in black while the noise enveloping the signals is visible in grey.

We conducted several experiments to evaluate different aspects of the performance of the denoising algorithms and present the quantitative results both in the tabular and the graphical form. Firstly, a comparison of various signal denoising algorithms have been carried out at multiple signal lengths *N*; ranging from 2^10^ to 2^14^. Next, the noisy signals at various input SNRs (i.e. −2 dB to 14 dB) have been denoised by the selected signal denoising algorithms for comparative study.

**Figure 7.**
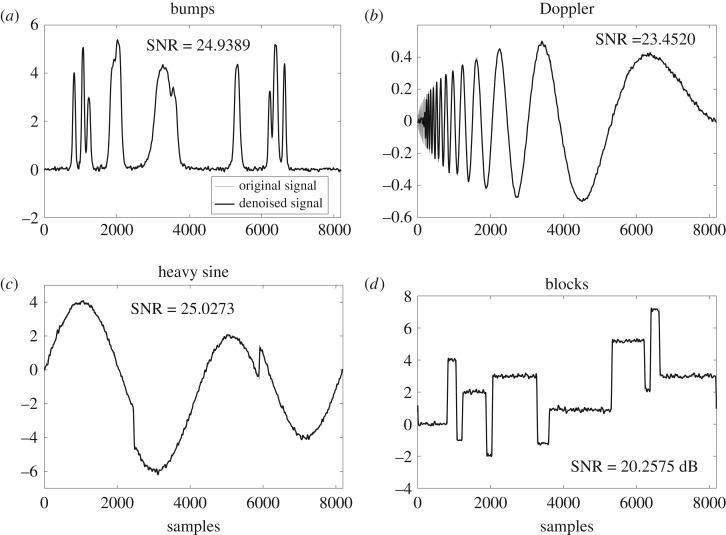
Denoised test signals using the proposed method: (*a*) bumps, (*b*) Doppler, (*c*) heavy sine, (*d*) blocks; for input SNR = 10 dB and input signal length *N* = 2^13^.

The qualitative aspect of the performance of the proposed method is demonstrated visually in figure 7, where the denoised versions of the noisy signals given in figure 6 have been shown. The comparison between the original signals in figure 6 and the denoised signals in figure 7 reveals that mostly the signals was recovered accurately, though a few artefacts are present in the regions where signal is varying slowly. Contrary to that, the high activity regions or the regions of sharp change were recovered completely in ‘bumps’, ‘Doppler’ and ‘heavy sine’ signals. The proposed method also closely recovers the ‘blocks’ signal despite having sharper discontinuities due to its piecewise constant nature.

#### 4.1.1. Signal length versus output SNR

Table 1 compares the output SNR/MSE of the proposed *DTCWT-GOF* against several state-of-the-art denoising methods at multiple signal lengths, ranging from *N* = 2^10^ to 2^14^. In this regard, four standard test signals (depicted in dark black in figure 6) were used to generate the results where each reported SNR value is the mean of SNR values obtained by denoising *J* = 1000 noisy realizations of the given signal corresponding to input SNR value 0 dB. Note that highest output SNR values for each noise level are highlighted in bold. The proposed *DTCWT-GOF-NeighFilt* comprehensively outperformed the selected state-of-the-art methods at all lengths for the signal ‘heavy sine’. In this case, the difference between the SNR/MSE values of the proposed *DTCWT-GOF-NeighFilt* and the second best method namely the *TI-DWT-GOF* was found to be considerably large and statistically significant which emphasizes the effectiveness of the proposed method.

**Table 1.** Performance evaluation, in terms of mean output *SNR*/*MSE*, of the proposed *DTCWT*-*GOF*-*NeighFilt* method against the comparative methods for various signal lengths *N* at an input *SNR* = 0 dB. Highest output SNR values for each noise level are highlighted in bold.

log_2_*N*	10	11	12	13	14
(*a*) *bumps*					
*BLFDR*	8.87/0.425	10.25/0.310	11.81/0.214	13.26/0.153	15.61/0.090
*TI*-*EMD*	9.57/0.360	11.17/0.247	12.86/0.168	14.55/0.113	15.40/0.249
*DTCWT*	8.63/0.444	**12.30/0.191**	13.81/0.135	14.08/0.126	14.14/0.124
*TVD*	10.03/0.402	11.18/0.192	12.11/0.195	13.16/0.155	12.95/0.145
*TI*-*DWT*-*GOF*	**10.80/0.735**	12.12/0.541	13.43/0.400	14.25/0.330	15.21/0.265
*DTCWT*-*GOF*-*NF*	8.34/0.473	11.94/0.207	**14.62/0.111**	**15.30/0.095**	**15.42/0.093**
(*b*) *Doppler*					
*BLFDR*	9.70/0.0093	10.43/0.0078	11.24/0.0065	13.56/0.0030	14.02/0.0034
*TI*-*EMD*	**10.44/0.007**	11.85/0.004	12.76/0.003	14.46/0.002	15.16/0.009
*DTCWT*	9.94/0.008	12.11/0.005	13.48/0.003	14.04/0.003	14.12/0.003
*TVD*	4.56/0.057	5.52/0.045	7.28/0.011	8.44/0.009	9.73/0.0053
*TI*-*DWT*-*GOF*	9.71/0.009	12.14/0.005	13.96/0.003	15.55/0.001	16.06/0.001
*DTCWT*-*GOF*-*NF*	9.76/0.009	**12.30/0.005**	**14.19/0.003**	**16.00/0.0027**	**16.08/0.0026**
(*c*) *heavy sine*					
*BLFDR*	11.51/0.707	11.95/0.622	11.81/0.6345	14.40/0.351	14.32/0.3551
*TI*-*EMD*	11.84/0.642	12.56/0.539	13.02/0.487	14.55/0.339	14.95/0.310
*DTCWT*	13.95/0.390	14.11/0.371	14.08/0.374	14.12/0.369	14.11/0.370
*TVD*	14.36/0.348	14.20/0.380	12.71/0.248	12.39/0.440	11.23/0.285
*TI*-*DWT*-*GOF*	12.96/0.493	14.86/0.310	15.43/0.263	15.69/0.240	16.31/0.223
*DTCWT*-*GOF*-*NF*	**14.96/0.270**	**15.05/0.307**	**15.74/0.280**	**15.92/0.277**	**16.77/0.268**
(*d*) *blocks*					
*BLFDR*	8.83/1.169	9.41/1.019	11.21/0.669	12.13/0.540	14.24/0.332
*TI*-*EMD*	9.93/0.898	11.01/0.703	12.65/0.478	14.02/0.348	**15.49/0.249**
*DTCWT*	11.26/0.661	12.29/0.520	13.06/0.435	13.58/0.386	13.81/0.366
*TVD*	**13.01/0.486**	**13.29/0.326**	12.38/0.443	12.31/0.370	10.75/0.211
*TI*-*DWT*-*GOF*	10.80/0.735	12.12/0.541	13.43/0.400	14.25/0.330	15.21/0.265
*DTCWT*-*GOF*-*NF*	10.74/0.746	12.54/0.492	**13.94/0.355**	**14.54/0.309**	14.93/0.282

For the test signal ‘blocks’, the proposed *DTCWT-GOF-NeighFilt* demonstrated superior performance for signal lengths *N* = 2^12^ and 2^13^ achieving statistically significant difference when compared against the second best method. While at *N* = 2^10^ and 2^11^, the *TVD* yielded best performance and also provided statistically significant difference against the second best *DTCWT*. The *TI-EMD* showed best results at length *N* = 2^14^, giving statistically significant difference over the second best method.

For the ‘Doppler’ signal, the proposed *DTCWT-GOF-NeighFilt* beats the other methods at higher signal lengths *N* ≥ 2^11^; however, it was observed that the difference between the highest SNR value (yielded via the *DTCWT-GOF-NeighFilt*) and second highest SNR value was not found to be statistically significant at *N* = 2^14^. Whereas, the *TI-EMD* yielded superior performance at *N* = 2^10^, while also observing statistically significant difference against the second best method. The *TVD*, in this case, failed to show the reasonable results as compared to the rest of the denoising methods.

For ‘bumps’ signal, the proposed method beats the rest of the denoising methods for lengths *N* ≥ 2^12^ with statistically significant margin against all methods. The *TI-DWT-GOF* performed best at *N* = 2^10^ with statistically significant margin over the *TI-EMD* which was the second best. The *DTCWT* provided best results at *N* = 2^11^ while the proposed method yielded third best performance in terms of output SNRs/MSEs.

#### 4.1.2. Input SNR versus output SNR

**Figure 8.**
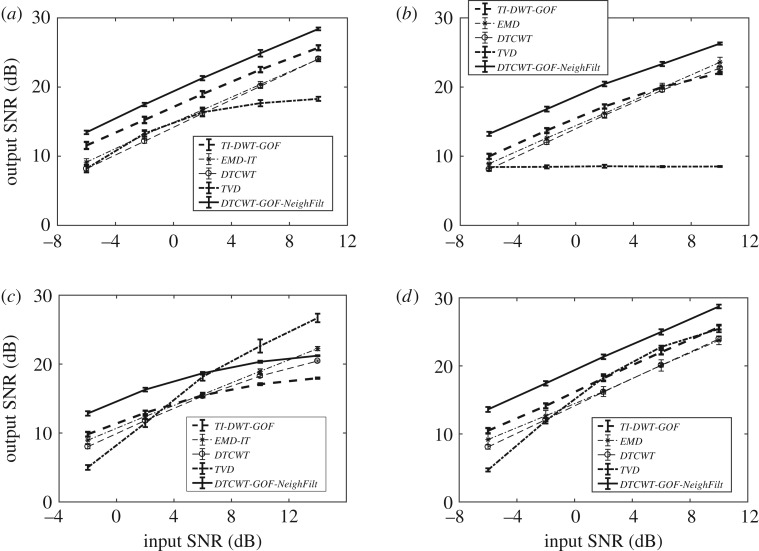
Output *SNR* versus input *SNR*: repeated simulation results obtained from applying the proposed and comparative denoising methods, on signal lengths *N* = 2^13^ for the test signals: (*a*) bumps, (*b*) Doppler, (*c*) heavy sine, (*d*) blocks.

We now demonstrate the performance of several denoising methods at various input SNR values for the four test signals. Figure 8 plots the error bars of the output SNR of various denoising algorithms for input SNR values ranging from −2 dB to 14 dB for the four test signals each of length *N* = 2^13^ (depicted in dark black in figure 6). The error bars help in visualizing the stretch (variance) of the output SNR values over *J* = 1000 realizations around the mean value for several denoising algorithms at each input SNR value.

The error bars in figure 8*c* are the result of repetitive experiments on the ‘heavy sine’ signal through the comparative where the proposed method comprehensively outperformed the state-of-the-art denoising methods for the whole range of input signal SNR. The *TI-DWT-GOF* stands second best for lower values of input signal SNR. While for higher values of input signal SNR, the *TVD* yields superior results. Here, the statistically significant differences were obtained for the best performing method (i.e. *DTCWT-GOF-NeighFilt*) against the second best method at each input SNR.

The error bars for the ‘bumps’ signal are shown in figure 8*a*. Here again, the proposed *DTCWT-GOF-NeighFilt* comprehensively beat the comparative denoising methods for the whole range of input SNR (i.e. −2 dB to 14 dB) where the statistically significant margin against the second best the *TI-DWT-GOF* was obtained. Similarly, in the error bars for the ‘Doppler’ signal given in figure 8*b*, the proposed method showed best results for all input SNRs (i.e. −2 ≥ SNR ≤ 14), yielding statistically significant margin against the second best *TI-DWT-GOF*.

Figure 8*d* showed the error bars plotted for the ‘blocks’ signal, where the *TI-DWT-GOF* shows best performance at higher input SNR values (i.e. 10 and 14 dB) while also yielding statistically significant result against the second best method. However, the proposed method yielded much superior as well as statistically significant ouput SNRs against the rest of the state-of-the-art methods at lower input SNRs (i.e. −2 dB to 6 dB).

Table 2 displays the output SNR/MSE values against a range of input SNR values, as measures to compare the performance of the selected state-of-the-art methods against the proposed method. All the SNR/MSE values listed in this table are mean of *J* = 1000 realizations. Note that highest output SNR values for each noise level are highlighted in bold. The salient feature of the proposed *DTCWT-GOF-NeighFilt* is that it beats the comparative methods for all signals with a considerably large margin. For ‘bumps’, ‘heavy sine’ and ‘Doppler’ signals, the proposed method yielded superior performance against all the comparative state-of-the-art methods at all input noise levels. Whereas, for the ‘blocks’ signal, the proposed method showed better denoising results for lower input SNRs while the *TVD* performed best at input SNRs = 10 and 14 dB. The proposed method outperformed the second best *TI-DWT-GOF* by a considerably larger as well as providing statistically significant margins. In fact, in most cases these margins lie between 20% and 30% of the second best which can be considered as significant improvement compared against the existing state of the art of one-dimensional signals.

**Table 2.** Performance evaluation in terms of mean output *SNR*/*MSE* of the proposed *DTCWT*-*GOF*-*NeighFilt* method against various comparative methods for a range of input *SNR* by conducting this experiment on four standard signals, each of length *N* = 2^13^. Highest output SNR values for each noise level are highlighted in bold.

SNR	−2 dB	2 dB	6 dB	10 dB	14 dB
(*a*) *bumps*					
*BLFDR*	5.59/0.898	10.78/0.273	15.85/0.085	20.80/0.027	25.02/0.010
*TI*-*EMD*	9.14/0.397	13.07/0.161	16.69/0.070	20.38/0.030	24.04/0.013
*DTCWT*	8.18/0.494	12.16/0.197	16.18/0.078	20.11/0.032	24.06/0.013
*TVD*	8.21/0.490	13.29/0.151	16.35/0.074	17.67/0.055	18.29/0.045
*TI*-*DWT*-*GOF*	11.55/0.228	15.24/0.097	19.00/0.041	22.52/0.018	25.69/0.008
*DTCWT*-*GOF*-*NF*	**13.44/0.146**	**17.21/0.061**	**21.28/0.024**	**24.89/0.010**	**28.40/0.004**
(*b*) *Doppler*					
*BLFDR*	5.94/0.022	10.93/0.0070	16.07/0.0021	21.02/6.8 × 10^−4^	25.15/2.6 × 10^−4^
*TI*-*EMD*	8.92/0.011	12.63/0.004	16.19/0.002	19.93/8.8 × 10^−4^	23.59/3.8 × 10^−4^
*DTCWT*	8.11/0.013	12.00/0.005	15.93/0.002	19.55/9.5 × 10^−4^	22.73/4.6 × 10^−4^
*TVD*	8.43/0.012	8.46/0.0099	8.55/0.010	8.49/0.0093	8.51/0.0009
*TI*-*DWT*-*GOF*	10.00/0.008	13.74/0.003	17.20/0.001	20.00/0.0008	22.04/0.0005
*DTCWT*-*GOF*-*NF*	**13.23/0.004**	**16.81/0.001**	**20.44/0.0008**	**23.33/0.0004**	**26.29/0.0002**
(*c*) *heavy sine*					
*BLFDR*	6.11/2.351	11.36/0.704	17.12/0.188	22.62/0.053	27.56/0.017
*TI*-*EMD*	9.17/1.172	12.67/0.523	16.22/0.230	20.06/0.095	23.76/0.040
*DTCWT*	8.08/1.485	12.10/0.589	16.16/0.231	20.10/0.093	23.96/0.038
*TVD*	4.71/3.201	11.91/0.605	18.16/0.155	22.79/0.049	25.43/0.030
*TI*-*DWT*-*GOF*	10.47/0.857	14.12/0.370	18.18/0.145	22.02/0.060	25.69/0.026
*DTCWT*-*GOF*-*NF*	**13.59/0.417**	**17.43/0.172**	**21.33/0.070**	**24.98/0.030**	**28.73/0.012**
(*d*) *blocks*					
*BLFDR*	5.03/2.778	9.80/0.925	14.37/0.324	18.56/0.123	21.75/0.059
*TI*-*EMD*	8.96/1.125	12.41/0.507	15.60/0.243	18.96/0.112	22.24/0.053
*DTCWT*	8.02/1.393	11.82/0.581	15.35/0.258	18.28/0.131	20.45/0.079
*TVD*	5.00/2.788	11.45/0.601	18.16/0.140	**22.63/0.051**	**26.70/0.011**
*TI*-*DWT*-*GOF*	9.82/0.920	12.94/0.449	15.48/0.250	17.08/0.172	17.97/0.141
*DTCWT*-*GOF*-*NF*	**12.84/0.458**	**16.29/0.206**	**18.67/0.119**	20.34/0.081	21.22/0.066

### 4.2. Experimental results on real signals

**Figure 9.**
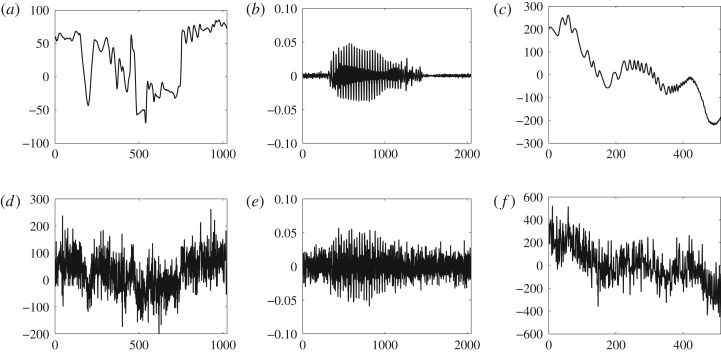
Real signals used for experimentation in this paper (*a*–*c*) along with their noisy versions corrupted by AWGN of 0 dB (*d*–*f*). (*a*) Tai-Chi signal, (*b*) speech signal, (*c*) float drift signal, (*d*) noisy Tai-Chi signal, (*e*) noisy speech signal and (*f*) noisy float drift signal.

In this section, we demonstrate the performance of the proposed method on real signals. To this end, the following real signals with varying inherent structure are employed for experimentation; a Tai-Chi sequence signal of human body motion, a speech signal and an oceanographic float drift signal. The Tai-Chi signal of length *N* = 1024 is a part of hexavariate recordings of human body movements in a Tai-Chi sequence, shown in figure 9*a*, obtained via two inertial three-dimensional sensors which were attached to left hand and left ankle [27]. The speech signal of length *N* = 2048, is a segment from the NOIZEUS database as shown in figure 9*b*, freely available at http://ecs.utdallas.edu/loizou/speech/noizeus. The oceanographic float drift signal of length *N* = 512, contains the float recordings of latitude drift of the water flowing through the Mediterranean sea which was recorded as part of the ‘Eastern Basin’ experiment [43] as shown in figure 9*c*. These signals were corrupted by adding the additive WGN of varying levels and were subsequently denoised via the comparative state-of-the-art methods along with the proposed *DTCWT-GOF-NeighFilt*. For this purpose, the *TI-DWT-GOF*, *TI-EMD* and *BLFDR* were selected as comparative state-of-the-art methods for experiments in this section.

**Figure 10.**
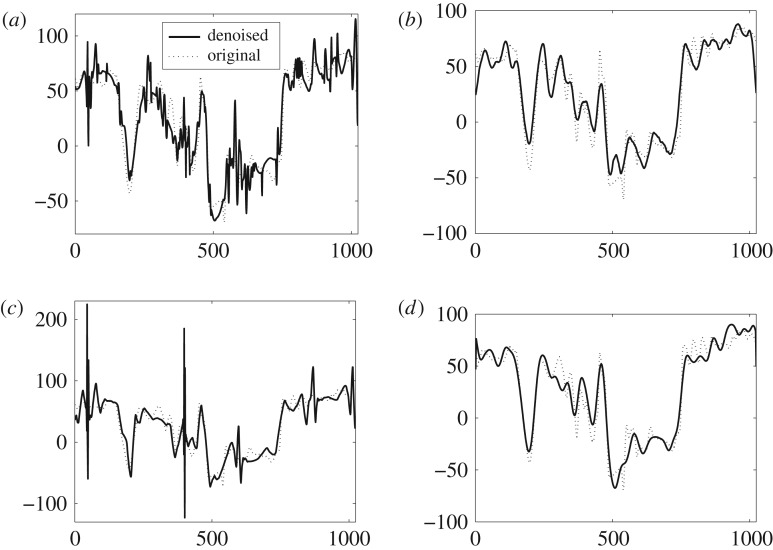
Visual denoising results on Tai-Chi signal corrupted by additive WGN of 0 dB via the comparative denoising methods. (*a*) *TI-EMD*, (*b*) *TI-DWT-GOF*, (*c*) *BLFDR* and (*d*) *DTCWT-GOF-NeighFilt*.

Figure 10 shows Tai-Chi signals obtained by denoising the noisy version of the Tai-Chi signal of SNR = 0 dB (depicted in figure 9*d*) through the comparative methods. In order to show how well the denoised signal resembles the original one, the original Tai-Chi signal is plotted along with each of the denoised versions in figure 10. It is evident from figure 10*d* that denoised Tai-Chi signal through the *DTCWT-GOF-NeighFilt* (plotted in dark black) closely follows the original one (plotted in dotted black). In fact, the *DTCWT-GOF-NeighFilt*-based denoised signal captures most of the details in the original signal unlike the other denoised signals shown in figure 10*a*–*c*. Second best visual results are shown by the *TI-DWT-GOF*, as shown in figure 10*b*, wherein a good estimate of original signal is recovered. The denoised signal via the *TI-EMD* in figure 9*a* contains too many undesired fluctuations which may be due to the false detection of noisy coefficients as desired signal. Similar kind of fluctuations, though larger in amplitude, are observed in the denoised signal via the *BLFDR* as shown in figure 10*c*.

**Figure 11.**
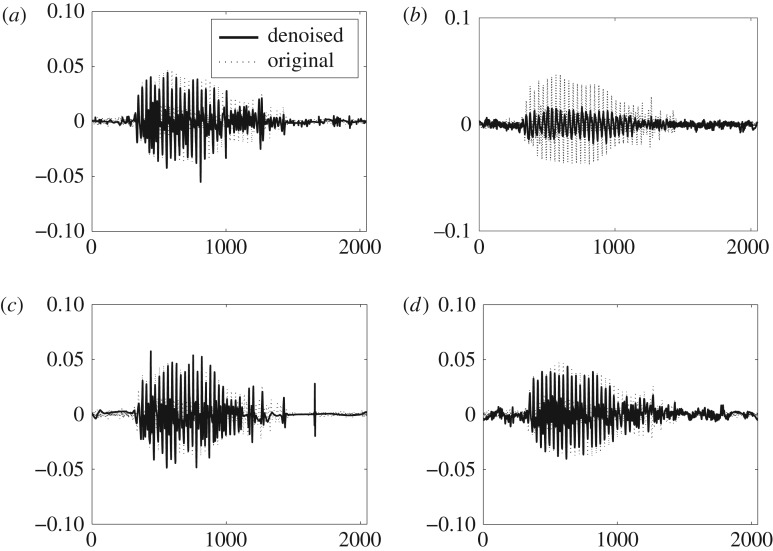
Visual denoising results on speech signal corrupted by additive WGN of 0 dB via the comparative denoising methods. (*a*) *TI-EMD*, (*b*) *TI-DWT-GOF*, (*c*) *BLFDR* and (*d*) *DTCWT-GOF-NeighFilt*.

Figure 11 shows visual results of comparative signal denoising methods obtained by denoising the noisy speech signal in figure 9*e* of SNR = 0 dB. Here, to visually depict the quality of the estimated signal via the comparative methods, original signal is plotted along with each of the denoised versions. It can be seen in the figure that the recovered speech signals through the *TI-EMD* and the *BLFDR* contain undesired artefacts which deteriorate the quality of the estimated speech signal as shown in figure 11*a*,*c* respectively. On the contrary, no artefacts are visible in the denoised signal by the *DTCWT-GOF-NeighFilt* whereby the estimated speech signal is in close resemblance with the original speech signal as shown in figure 11*d*. The denoised signal by the *BLFDR* seems to miss the details in later part of the sound burst apart from the occasional fluctuations (artefacts) throughout the signal. Owing to undesired fluctuations, though comparatively lesser in magnitude than the results shown by the *BLFDR*, the *TI-EMD* presents a decent estimate of the original signal which is second only to the proposed method. The *TI-DWT-GOF* gives worst denoising results as shown in figure 11*b* where most of the significant information is lost.

#### 4.2.1. Input SNR versus output SNR

In this experiment, comparative state-of-the-art signal denoising methods were operated on real-world data which were corrupted by additive WGN for comparison with the proposed *DTCWT-GOF-NeighFilt*. The quantitative results of this experiment are presented in table 3, where output SNR/MSE values of the denoised signals are reported by denoising their noisy versions at input SNR =−5 dB, 0 dB, 5 dB. Each output SNR/MSE value in table 3 is the mean of *J* = 1000 repetitions. The statistical significance of the best performing method is also verified at 5% significance level through Student's *t*-test against the second best result in order to validate the quantitative results.

**Table 3.** Performance evaluation of comparative signal denoising methods on real signals in terms of mean output *SNR*/*MSE* values against the input SNR =−5 dB, 0 dB, 5 dB.

input SNR	−5 dB	0 dB	5 dB
(*b*) *Tai-Chi Signal*			
*BLFDR*	4.13/1.0 × 10^3^	9.37/304	14.44/94.7
*TI*-*EMD*	6.14/645	10.95/213	15.16/79.9
*TI*-*DWT*-*GOF*	6.66/567	12.21/158	14.00/104
*DTCWT*-*GOF*-*NeighFilt*	**9.84/275**	**13.24/124**	**15.38/75.8**
(*a*) *speech signal*			
*BLFDR*	1.50/9.3 × 10^−5^	5.20/3.9 × 10^−5^	8.87/1.7 × 10^−5^
*TI*-*EMD*	3.35/6.1 × 10^−5^	7.50/2.3 × 10^−5^	**11.20/1.0 × 10^−5^**
*TI*-*DWT*-*GOF*	0.004/1.3 × 10^−4^	0.05/1.3 × 10^−4^	0.09/1.2 × 10^−4^
*DTCWT*-*GOF*-*NeighFilt*	**3.46/5.9 × 10^−5^**	**7.70/2.2 × 10^−5^**	11.04/1.0 × 10^−5^
(*c*) *float drift signal*			
*BLFDR*	2.89/7.1 × 10^3^	8.65/1.9 × 10^4^	12.69/737
*TI*-*EMD*	5.55/3.9 × 10^3^	9.84/1.1 × 10^3^	**14.34/503**
*TI*-*DWT*-*GOF*	7.85/2.2 × 10^3^	9.20/1.6 × 10^3^	10.79/1.1 × 10^3^
*DTCWT*-*GOF*-*NeighFilt*	**8.07/2.1 × 10^3^**	**10.06/1.3 × 10^3^**	11.14/1.0 × 10^3^

From table 3, it is clear that the proposed *DTCWT-GOF-NeighFilt* demonstrates superior results for the Tai-Chi signal at all input noise levels. The *TI-DWT-GOF* shows second best performance at input SNR =−5 dB and 0 dB while the *TI-EMD* gives second best results at input SNR = 5 dB. It was observed that the mean output SNR values yielded by the proposed method maintain statistically significant difference against the second best methods at all noise levels. Despite being last at input SNR −5 dB and 0 dB, the *BLFDR* shows competitive results at input SNR = 5 dB.

For speech and float drift signals, the proposed method shows best results in terms of mean output SNR/MSE values at input SNR =−5 dB and 0 dB. At both of these noise levels, results yielded through the proposed method demonstrate statistical significance when compared against the second best method for the oceanographic float drift signal. However, for speech signal, the statistical significance is observed only at input SNR = 0 dB.

The TI-EMD outperforms all other methods at input SNR = 5 dB for both of the float drift and speech signals. For the float drift signal, the TI-EMD demonstrates statistical significance against the second best *BLFDR* at input SNR = 5 dB while the proposed method ranks third in terms of mean output SNR values. Contrarily, for the speech signal, the proposed method ranks second to the best performing method *TI-EMD* whereby no statistically significant distance was observed between the two methods.

## 5. Conclusion

In this paper, a novel signal denoising algorithm has been proposed which employs the GOF test on the complex wavelet coefficients, obtained via the DT-CWT, in order to classify the coefficients as signal and noise. Subsequently, a novel neighbourhood filtering technique is introduced to detect false noise classifications in the previous step. The detected false noise classifications are restored to original values while true noise classifications are discarded. Within the GOF test, the statistics based on EDF have been employed to estimate the similarity between the wavelet coefficients corresponding to signal and those belonging to noise.

The experimental results have been shown at synthetic as well as real signals whereby the proposed method comprehensively beats the comparative signal denoising methods. The performance of the proposed method has been particularly better at higher noise levels against the state-of-the-art denoising methods, where the margin by which proposed method beats the second best has been quite significant.

The proposed method has been designed to work for additive Gaussian noise distributions with zero mean and arbitrary variance. One possibility for future work could be to extend this framework to non-Gaussian distributions and/or to Gaussian distribution with arbitrary mean and variances.

## Footnotes

1 A part of this work was presented at IEEE International Conference on Digital Signal Processing (DSP), August 2017 at London, UK [33].

## Data accessibility

Our data are deposited at Dryad Digital Repository: https://doi.org/10.5061/dryad.7j699nc [44].
